# Ten simple rules to complete successfully a computational MSc thesis project

**DOI:** 10.1371/journal.pcbi.1012756

**Published:** 2025-01-28

**Authors:** Edoardo Saccenti, Cristina Furlan

**Affiliations:** Laboratory of Systems and Synthetic Biology, Wageningen University & Research, Wageningen, the Netherlands; Carnegie Mellon University, UNITED STATES OF AMERICA

## Abstract

The thesis project is an essential step to obtain an MSc degree. Within STEM and Life Sciences disciplines, computational theses have specific characteristics that differentiate them from wet laboratory ones. In this article, we present *Ten simple rules* to direct and support Master students who are about to start a computational research project for their Master thesis. We begin by recommending defining the personal learning goals for the project; we then highlight specific pitfalls that computational students might encounter during their work, such as procrastination by computation or wasting time while attempting to reinvent computational tools. We provide the students a series of suggestions on how to work following FAIR principles, learn new computing languages, and think ahead for computational challenges. We hope that these 10 simple rules will provide Master students with a framework for the successful completion of their computational thesis.

## Introduction

Completing a Master thesis project requires a significant investment of time, effort, and resources, but when completed successfully, it can also be a highly rewarding experience, providing skills and knowledge that are valuable for the student’s future academic or professional pursuits.

In the *Ten rules* series much attention has being paid to graduate students, with several set of rules dealing with different stages and aspects of the PhD trajectory: for aspiring PhD candidates [1]; for the selection of the right supervisor [2]; for successfully completing the PhD [3] taking into account its many facets [4–6]. Most of these rules address challenges and difficulties that graduated students may encounter, but little or no attention has been given to the research projects that serve as a stepping stone towards a PhD trajectory and the job market: the MSc thesis project.

While there are some similarities between the MSc and PhD thesis work (both entail performing original research and the writing of a report/dissertation), there are also many differences. The MSc thesis project is an educational activity where the student is required to use, apply, and expand the knowledge and skills acquired in previous courses: as such, an MSc thesis work is usually short (6 months to 1 year, depending on the country). In contrast, a PhD trajectory is an extended endeavour usually restricted to 3 to 4 years in most European countries as opposed to the USA, where the median time to PhD varied between 6 and 10 years [7]. There are also other defining aspects, which are implicitly reflected by the fact that supervision of MSc and PhD students are diverse processes [8] with diverse expectations.

Whatever the field of research, completing an MSc thesis requires self-reflection, choosing the right project and supervisor, implementing feedback, and effective time and research management skills.

For what concerns STEM (science, technology, engineering, and mathematics) and Life Sciences thesis projects, there are practical and organisational differences between performing lab-based or computational research, that have been made evident during the COVID pandemic that has caused an almost total halt of (non-COVID) lab-based research activity [9], while, with some adjustments, was possible to manage computational research remotely.

Nonetheless, thesis projects which focus on computational research, come with their specific challenges and pitfalls: we present here a set of 10 rules (or better suggestions) specifically dedicated to Master students who, at the final stage of their MSc education trajectory, are about to start their thesis research project.

In these 10 rules we directly address the students, offering practical advice and suggestions and we also provide references for those readers who may be interested in broadening their knowledge about education. We also refer to several other *Ten rules* articles, providing an entry point to many resources and guidelines that a student can find useful.

Rules 3 to 8 and 10, deal specifically with the case of computational projects, while with rules 1, 2, and 9 we address some critical aspects that all MSc students, irrespective from the type of research they will be performing, may face. Rule 8 deals with good research practice, FAIR principles and Open Science, and it is certainly of interest also for non-computational research.

## Rule 1: Define the learning goals of your thesis

A Master thesis project is an educational and training activity where you will be able to apply the skills and competences that you have acquired during your studies. Before starting (to look for) a thesis project, it is important that you define your personal learning goals, that are the intended outcomes and desired accomplishments defining the knowledge, new abilities, competencies, and skills that you want to acquire during the thesis work. You should reflect on this before starting to look for a thesis project and before contacting a thesis supervisor, because starting your MSc thesis project with defined and attainable goals can increase your academic performance [10].

Usually, a Master thesis project is already defined by the supervisor (and, incidentally, choosing the right supervisor is fundamental, see [2]), so be sure that the project aligns with your learning goals and interests. Made them clear to your prospective supervisor and be open about your intended goals. Defining learning goals is a defining moment in your thesis trajectory and involves a mix of planning for the future (see **Rule 2**) and self-reflection (see **Rule 3**).

Ultimately, having well-designed learning goals can help you to stay motivated and engaged throughout the research project [11].

## Rule 2: Think about your next career step

Completing your thesis project is not only a requirement to obtain an MSc degree but can be a first step towards a career in scientific research. Your study programme may require an additional internship in another research institute or university or a company placement. You may be interested in working in industry or you may even be interested in pursuing a PhD after you graduate. If you already know your next career step you should keep this in mind while choosing the thesis project. Or else make sure to use the abovementioned formative experiences to explore different career paths and engage in a project that provides new skills and challenges from your current thesis.

A company internship is a common requirement for many MSc in STEM in European universities (and for MPhil or MRes in UK). In general, you must arrange it on your own, and you must plan well in advance. Useful questions to ask yourself are: Do I already have an interest for a specific subject or research area? Am I committed to work on the long term on a specific topic? Are there knowledge gaps that I want to explore with the next experience? Is there a specific company, type of industry, or research institution or group that I would like to get acquaintance with? In this case, you should choose a thesis project that aligns with the company interests. Consult these 10 simple rules for (prospective) interns [12,13]. In addition, the rules presented in [14] offer a software company perspective on hosting and hiring an intern and contains useful insights also for the applicants.

If you wish to do a PhD after your MSc (you can read these rules for aspiring graduate students [1,2]), be aware that different countries organise PhD in different ways: in some countries you can start your PhD at any time (like in the Netherlands), in other there are fixed starting dates as some PhD programmes start in the fall (like in Italy), in many countries or research institutes you must apply to a PhD School and you will be facing a structured procedure with strict deadlines and requirements (e.g., Graduate Record Examinations, English tests, VISAs, and a many bureaucratic requirements). In any case, applying for a PhD is time consuming, and you may have to do it while working on your project.

At the beginning of your thesis, discuss the possibility of allocating time for this in your research schedule: planning for the future should not interfere with or delay your thesis work; in some circumstances you may need to be open to face a gap semester (or even a year) before finding the right position. By talking to your supervisor, you will not only be given advice on your choice to continue with a PhD but also the possibility of exploring opportunities within their research team, department, or their collaboration network.

Whether you plan a future in academia or in industry as a computational graduate, being able to showcase your work can increase the chances of landing an internship in a good company, research institute or university and a position as company employee or PhD candidate afterwards. You can do this by creating a portfolio or a Git repository (see **Rule 8** on working FAIRly) that can be shared with prospective employers to support your applications.

## Rule 3: Self-assess your own computational competencies

Self-assessment of computational skills can be a significant barrier for you as a student at the beginning of a research trajectory [15]. It is known that it is difficult to assess our own competences: this is the so-called Dunning–Kruger effect [16] according to which people with limited knowledge on a certain topic tend to be unaware of their limitations and misestimate their own competences. In other words, you may think you have excellent programming skills or knowing a lot about a certain computational topic, but this may be the case because you do not have enough experience or information to gauge efficiently how much you really know and how much may be needed to accomplish your research task.

You need to have an idea of what computational skills are required, and at which level you should master them, to start working on your project, because if you do not have such skills you will need to develop and acquire them while working on your project. This can not only hamper research progress but also can be source of stress, loss of motivation and self-esteem which will negatively affect the training process that an MSc thesis is supposed to embed.

For this reason, before the start of the thesis, you must clearly discuss with your (prospective) supervisor which computational skills are required and at which level of proficiency: then use the suggestions in Rule 4 to address your shortcomings. It may be extremely helpful to talk with other students working on related projects in the same research groups. Finally, if you realise that you do not have the skills required and you do not have the time or the opportunity for acquiring them, then it might be wiser to look for a different and more suited research project.

## Rule 4: Prepare for the computational challenges ahead

**Fig 1:**
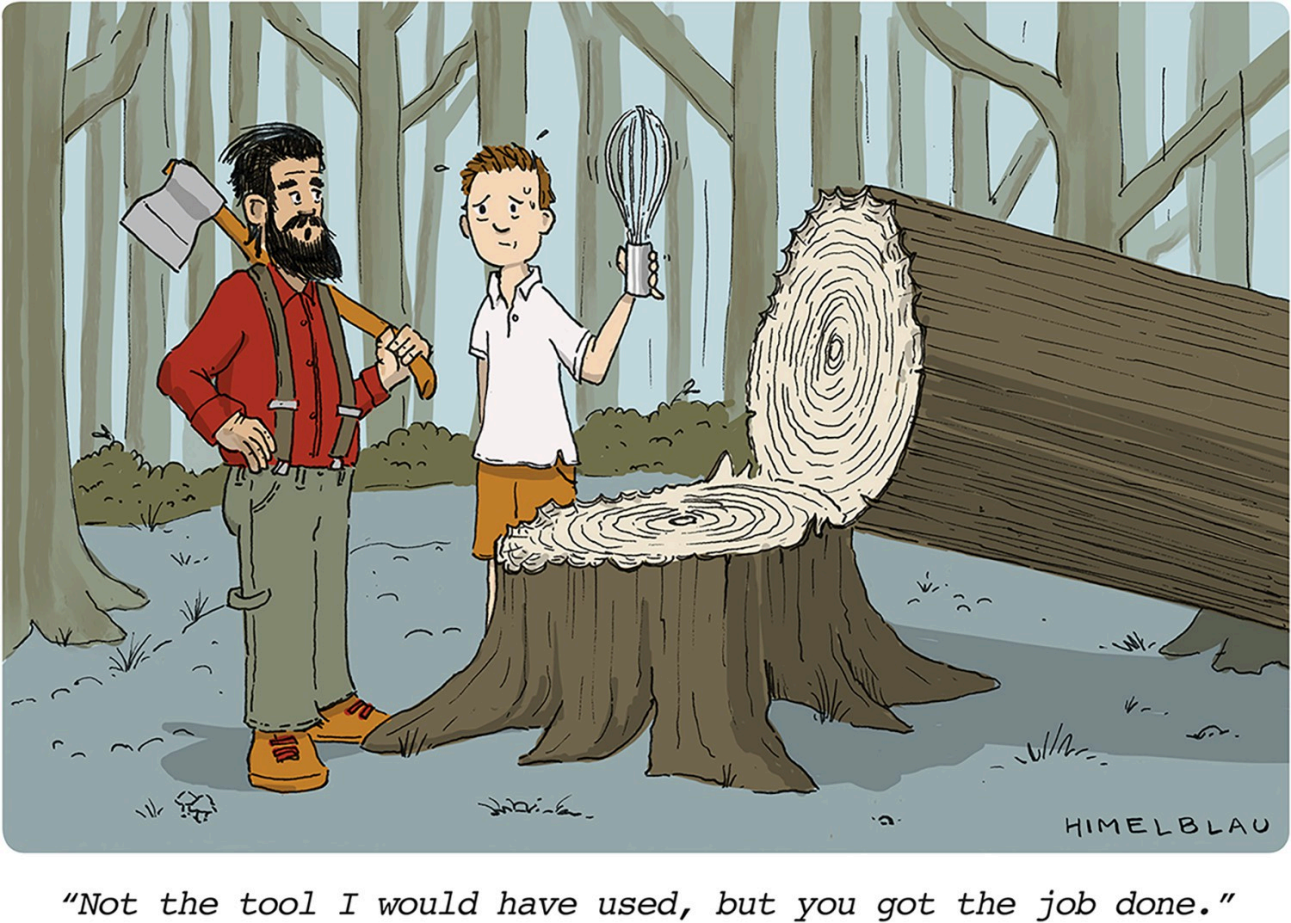
The choice of the right tool to accomplish a given task is often the key to success: do not “reinvent the wheel!” Cartoon by Ed Himelblau (www.himelblau.com; reproduced with permission of the author).

As a budding computational scientist, you will have the first taste of what it means to use computational tools to solve real-life problems and to conduct research. Everybody has their own preferred programming language and tools, but “If the only tool you have is a hammer, it is tempting to treat everything as if it were a nail” [17] (see Fig 1). Mastering only one programming language or set of computational tools will lead you to look at the problem from a limited or biassed perspective. Start expanding your set of computational skills before you start your thesis project: it will help to tackle challenges that may (and they will!) arise during your research project.

Many departments have a selection of prerequisite courses to ensure a student can start with a solid foundation on the topic. If a specific programming language is required for your project, make use of the infinite resources available online like free tutorials and courses to learn it. There are many online educational providers (e.g., Coursera, edEx, Udemy, DataCamp) and companies (e.g., Google, Microsoft, OpenAI) that can award you (micro)credentials and certificates of acquired skills that you can also show in your CV when looking for a job outside academia (see also **Rule 2**).

Nowadays, you can take advantage of generative AI tools to help you coding (coding productivity and syntax assistance) so that you can balance the effort you put in learning the new programming language with the time constraints of your project. However, you are always responsible of verification and rigour of the help you get from such tools [18]. Moreover, learning a new programming language is like learning a new language in adulthood [19]: the more you know, the easier it is to learn a new one!

## Rule 5: Do not reinvent the (computational) wheel

You may find that some computational tools you need to perform a certain task or analysis for your projects are not available in your preferred programming language. In these situations, do not “reinvent the wheel:” do not attempt to reimplement existing tools which are working and validated just to remain in your programming comfort zone. For this, it is important that you are familiar with more than 1 programming language (see **Rule 4**). In many cases, it is all about choosing the right tools for the job (see **Fig 1**)!

Within the limited time of your MSc thesis project, re-coding a tool or translating scripts to other programming language is a huge waste of time, time that you are diverting from the main tasks of your project. Plus, in most cases, there is no point in trying to replicate (usually with inferior results) something that other researchers may have spent years developing (if re-coding or re-developing a tool is not the goal of your thesis).

We have known students who have tried to re-code a Python ODE solver (reason: “Because I can do better.” See **Rule 3**.) or to implement in Bash shell program and command language a set of functions already existing in FORTRAN (reason: “I am not too familiar with FORTRAN.” See **Rules 3** and **4**.). Experience shows that this is not the most efficient and effective way of doing computational research.

## Rule 6: Start writing on day 1: Avoid procrastination by computation

**Fig 2:**
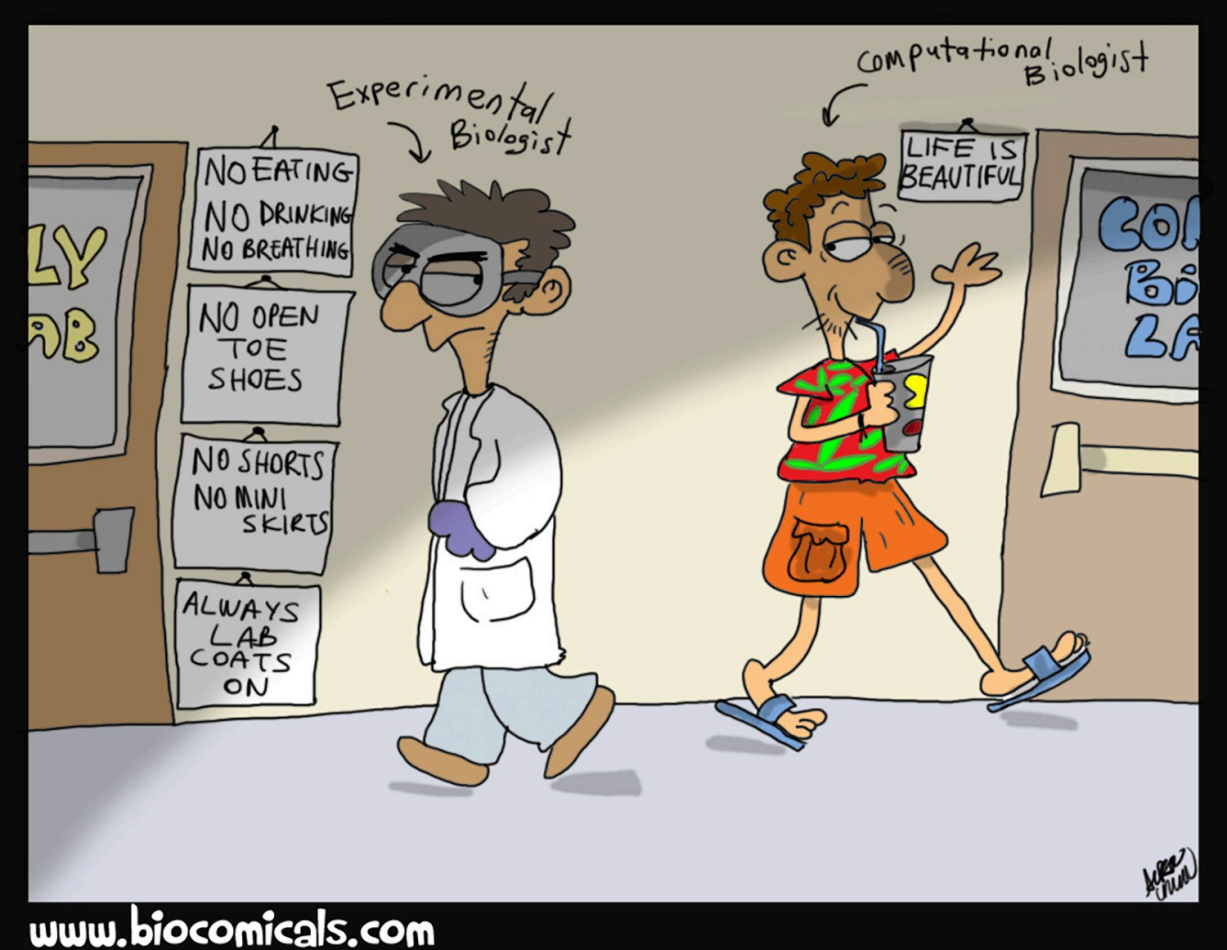
The life of the computational student as opposed to the experimental students is not as carefree as it seems. The risk of falling into procrastination by computation is always present. Cartoon by Alper Uzun (www.biocomicals.com; reproduced with permission of the author).

As a computational scientist you have higher freedom in organising your research as you are less constrained by experimental work and presence in the lab (see **Fig 2**): use this freedom wisely! It is quite easy to fall into the trap of delaying the start of the writing process by engaging in unnecessary computational experiments. This is what we call *procrastination by computation*. For many students, the writing of the thesis report is the less appealing and interesting part of the thesis experience, and keeping running computation is a good and plausible excuse to delay the writing.

You need to set yourself some boundaries with respect to computational work that you can engage with and the time you can spend on it. A well-thought research plan can help you with this. Problems in planning usually result in wasted time and lack of progress [20], while efficient time planning associates positively with academic achievement [21,22].

Prepare a time schedule and a list of deliverables to help you stay organised and focused throughout the thesis project: for this you can make a project plan using tools such as a Gantt chart [23]. If possible, based on the size of your data and the tools you are going to use try to estimate the computation required by the analysis you are going to perform: this will provide an indication of how long it will take you to generate your results and you can use this information for your planning.

## Rule 7: Attend scientific, code-reviews, thesis ring, and peer-meetings

You can receive great support for your research and thesis writing by participating in peer meetings, in particular attending thesis rings (thesis circles) [24] and code review-meetings. These forums give you the opportunities to interact with your peers, receive feedback on your work, and expand your knowledge and receive emotional support [25] and can positively affect your performance [26,27].

As a computational scientist, it is particularly important that you discuss your computer code and your ideas for computational solutions with your peers [28]. There are many advantages to be gained from code review meetings, from knowledge and skills development to enhanced quality of your output [29] and, of course, the chance of detecting bugs and errors [30].

Participating in these activities will help you to develop communication, people and soft skills (for instance, by practising presentation skills). These activities provide you with the opportunity of receiving feedback on your work from a diverse range of perspectives: instead of receiving feedback from only your supervisor, you will be exposed to a wider range of opinions and critiques.

Receiving feedback is a part of the creative process [31] that can be learnt by capturing and retaining new ideas, engaging in difficult tasks, expanding the skill set you have from your study, and seeking new stimuli [32].

Embracing the various activities of academic life can be seen at times as a hindrance to actual work on the scientific project especially when you can work from home for your project. As a student, you may not see the added value. However, departmental group meetings, seminars by local and international experts, and journal clubs will allow you to learn from experienced scientists by gaining insights into their reasoning and creative processes [25,33].

## Rule 8: Work FAIRly

In computational research is fundamental that code (mathematical models, code for statistical analysis, software, and analysis protocols) and data are managed and shared in a way that is open, transparent, and useful to others [34]. You must perform research according to the FAIR (Findable, Accessible, Interoperable, and Reusable) principles [35] which are the cornerstone of responsible research [36].

Your research should be FAIR by design, following a code and data management approach in which information is recorded at the same time of creation of your code or generation of your data to maintain provenance of your code, models, and data [37].

This goes hand in hand with keeping an electronic (e-)lab-journal: it is important that you learn how to maintain a good laboratory notebook [38]. This will make you involved with the project from day one and not only once the data are produced and delivered to you for analysis.

To work FAIRly, you can make use of code repository services like SourceForge, Bitbucket, GitLab, and GitHub [39,40]. There are also worksheets that combine the possibility of storing and running snippets of code, results of an analysis, associate text, and illustrations such as Jupyter Notebook, R markdown, Colab notebook (see, for instance [41]).

Following FAIR principles will ensure that your code and results can be easily retrieved and used by others in the future, giving more visibility and credit to your thesis work (see also **Rule 2**).

## Rule 9: Publish, do not perish

A research project ought to generate new findings and new knowledge, and scientists publish their research in scientific journals to share their discoveries. It may happen that your thesis work and the results that you have produced are of enough quality to contribute to an article and thus be published.

If you wish to stay in academia and pursue a PhD degree or plan to land a research job in a company, a published paper with your name on it is a well-recognised proof that you can conduct research (see **Rule 2**): discuss with your supervisor about the possibility of publishing your thesis work when talking over a possible thesis project because you will need to make some agreements on how to achieve that.

An MSc thesis is usually around 6 to 12 months, depending on the country: this means that very likely you may have to commit some time to keep working on finalising analysis and writing a paper (in most cases, this will happen in close interaction with your supervisor) after the end of your thesis, possibly while looking for a job or applying for a PhD position (again see **Rule 2**). We personally have encountered this situation several times, and having a paper published from MSc project is not uncommon: the research presented in references [42–48] originate from MSc student’s computational thesis projects in our group.

However, manage your expectations: despite your efforts and hard work your thesis may not result in a publication, since luck and opportunities also play a role in science and research careers [49].

If your thesis is just the last step before you graduate and leave the research world (for whatever reason) and have no interest in seeing your research published, be clear on this: your supervisor, another MSc student, or a PhD candidate may like or need to take over your research to finalise it. In this case, you need to be sure that all your code and results are accessible and understandable to other people: you can accomplish this by working FAIRly, as described in **Rule 8.**

It may also happen that even if your results are interesting, your supervisor has no interest in publishing them, no matter what. Ask about this to prospective supervisors: you should take their answer in consideration when deciding on which supervisor (and project) to choose among several (see **Rule 1**).

In any case, make clear agreements about the inclusion of your name on the author list (and in which position) of possible publications that might arise from your work, because a lack of clarity may lead to conflicts which can easily escalate to unpleasant disputes: scientists tend to be very sensitive on this topic [50].

## Rule 10: Switch from executing code to solving problems

If your thesis project addresses a Life Science problem, the overarching goal is usually to understand how Nature works. In this endeavour, performing computational research does not stop with writing good code for analysis. Writing and executing computational codes is often the easiest part and just the starting point. The challenging part is to reflect critically on the results you have obtained. You gather the evidence from observations, (computational) experiments, or readings and through interpreting, understanding, applying, and synthesising you make explicit and reasoned judgements [51]. To develop critical thinking, you should identify the different angles of the problem, compare, and contrast different views.

Critical thinking is a crucial skill to acquire to make the switch between executing and creating valuable knowledge and content for your research and for your thesis. Keep always in mind the biological problem you are trying to solve.

In other words: learn to always question the results of your computer code! If the results look too good to be true, they usually are not! Do not be afraid to challenge your own work!
